# Severe Polyhydramnios Associated With Antenatal Bartter Syndrome

**DOI:** 10.7759/cureus.109976

**Published:** 2026-05-31

**Authors:** Dynora Pierre-Louis, Iliza Minaya, Sohni Pathan, Jordan Taylor-Bentley, Kortnie Pryor

**Affiliations:** 1 Obstetrics and Gynecology, HCA Healthcare, Margate, USA; 2 Medical School, Nova Southeastern University's Dr. Kiran C. Patel College of Allopathic Medicine, Davie, USA

**Keywords:** amniotic fluid index (afi), antenatal bartter syndrome, polyhydramnios, salt-wasting, single deepest vertical pocket (sdvp)

## Abstract

Polyhydramnios is defined as an abnormal increase in amniotic fluid volume and is most commonly diagnosed during the second or third trimester of pregnancy. Although the majority of cases are idiopathic, polyhydramnios may also be associated with maternal conditions, fetal structural anomalies, and rare genetic disorders. We present the case of a 35-year-old G3P1102 patient at 27 weeks and 2 days of gestation who presented with preterm premature rupture of membranes (PPROM) and preterm labor. Obstetric ultrasonography demonstrated severe polyhydramnios with an amniotic fluid index (AFI) of 57.66 cm in the setting of a structurally normal fetus. In the postnatal period, the neonate developed brisk diuresis and electrolyte abnormalities, raising clinical suspicion for antenatal Bartter syndrome. Additional neonatal evaluation demonstrated renal salt wasting and hypercalciuria, findings supportive of this diagnosis. This case highlights the importance of considering antenatal Bartter syndrome in the differential diagnosis of severe unexplained polyhydramnios, particularly in the setting of a structurally normal fetus, as early recognition may facilitate perinatal planning and potentially improve neonatal outcomes.

## Introduction

Polyhydramnios is an abnormal increase in amniotic fluid volume diagnosed by ultrasonography during the second or third trimester of pregnancy, typically between 24 and 30 weeks’ gestation [1]. It is defined by a single deepest vertical pocket (DVP) ≥8 cm or an amniotic fluid index (AFI) ≥25 cm [2,3]. Polyhydramnios occurs in approximately 1-2% of pregnancies [2]. Although many cases are idiopathic, a broad range of maternal, fetal, and placental conditions may contribute to its development. Common etiologies include maternal diabetes mellitus, fetal structural anomalies, placental abnormalities, and rare genetic disorders. Polyhydramnios may result from pathologies that impair fetal swallowing, reduce GI fluid reabsorption, or increase fetal urine production [3]. Additional causes include twin-twin transfusion syndrome, fetal macrosomia, cardiac and renal abnormalities, alloimmunization, and congenital infections [2,3]. Less commonly, polyhydramnios may arise from fetal osmotic diuresis in high-output cardiac states or hydrops fetalis [3]. Despite extensive evaluation, a substantial proportion of cases remain without an identifiable cause.

Polyhydramnios may be categorized as mild (AFI 24.0-29.9 cm or DVP 8-11 cm), moderate (AFI 30.0-34.9 cm or DVP 12-15 cm), or severe (AFI ≥35.0 cm or DVP ≥16 cm) [2]. Increasing severity is associated with greater risks of preterm premature rupture of membranes (PPROM), cord prolapse, placental abruption, preterm delivery, perinatal mortality, and delivery of small-for-gestational-age infants [2]. Because moderate-to-severe polyhydramnios is more frequently associated with underlying pathology, a detailed maternal and family history and targeted fetal USG are warranted [3]. In severe cases, maternal symptoms, including dyspnea and abdominal discomfort, may occur. Uterine overdistention may also contribute to preterm labor and an increased risk of membrane rupture. Therapeutic amnioreduction may be considered for symptomatic relief [2,3].

In rare instances, early-onset severe polyhydramnios in the setting of a structurally normal fetus may represent the initial manifestation of antenatal Bartter syndrome, a renal tubular disorder characterized by impaired chloride transport in the thick ascending limb of the loop of Henle, resulting in fetal polyuria, severe polyhydramnios, and an increased risk of preterm delivery [1,4]. This case report highlights severe polyhydramnios occurring in the absence of identifiable maternal, fetal, or placental abnormalities, raising clinical suspicion for antenatal Bartter syndrome and underscoring the importance of considering rare etiologies when evaluating polyhydramnios in an otherwise structurally normal fetus.

## Case presentation

A 35-year-old G3P1102 at 27 weeks and 2 days of gestation was admitted for evaluation of preterm labor and PPROM. Her pregnancy was complicated by severe polyhydramnios and a history of two prior cesarean deliveries. Prenatal USG performed by Maternal-Fetal Medicine (MFM) demonstrated severe polyhydramnios with an AFI of 43.56 cm, normal fetal growth, and no major structural abnormalities. Amniocentesis was recommended for further evaluation of the severe polyhydramnios; however, the patient declined.

**Figure 1:**
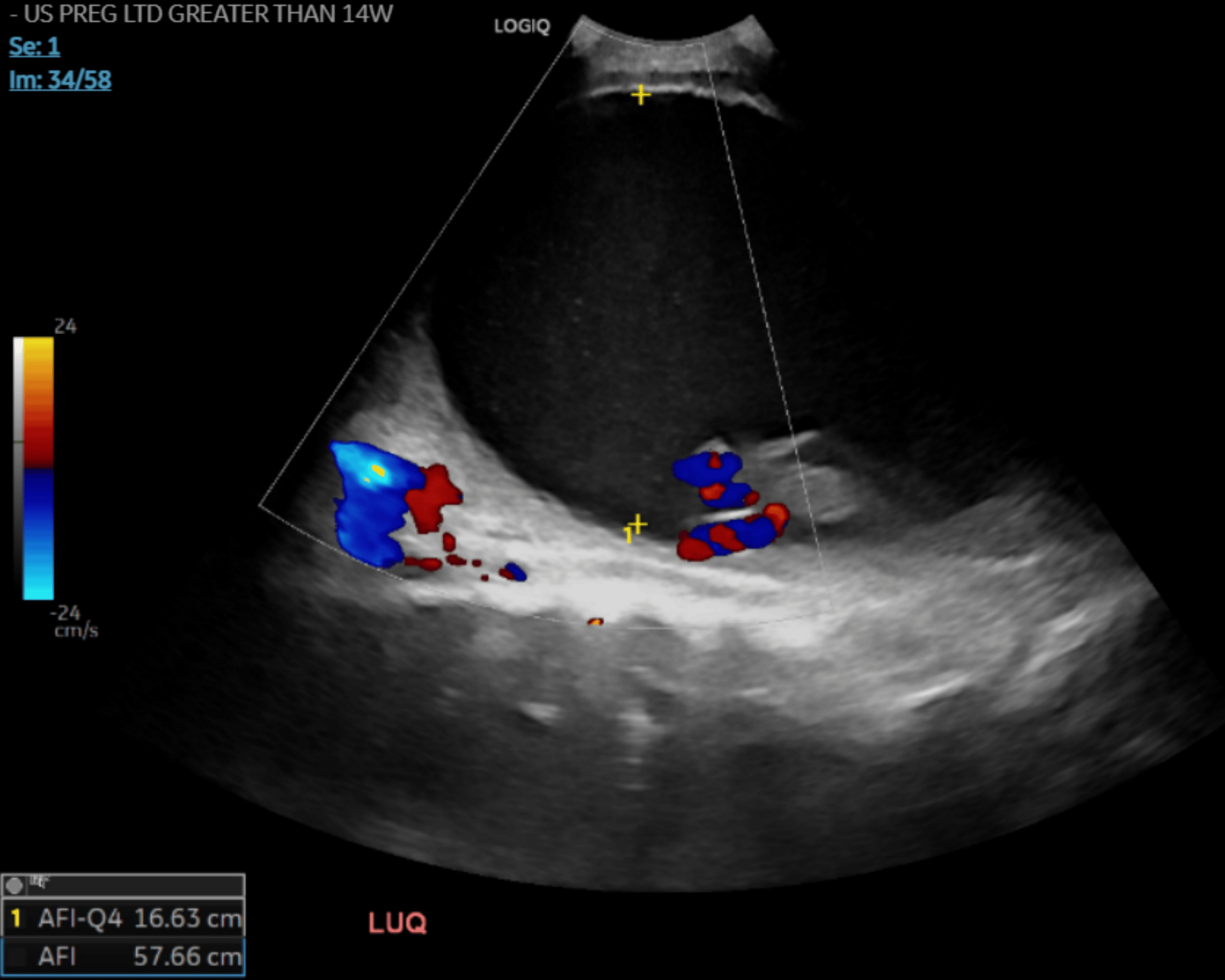
Obstetric ultrasound demonstrating severe polyhydramnios, with an amniotic fluid index of 57.66 cm. AFI: Amniotic fluid index.

Upon admission, repeat USG demonstrated worsening polyhydramnios with an AFI of 57.66 cm (Figure 1). The patient was started on latency antibiotics, including IV ampicillin 2 g every 6 hours and a single dose of azithromycin 1,000 mg orally; magnesium sulfate for fetal neuroprotection, with a 4 g loading dose followed by a 2 g maintenance infusion; antenatal corticosteroids for fetal lung maturation, with intramuscular betamethasone 12 mg administered every 24 hours for two doses; and tocolytic therapy with oral indomethacin 50 mg every 6 hours. Despite these interventions, preterm labor progressed, and she subsequently underwent repeat cesarean delivery at 27 weeks and 4 days of gestation.

Postnatal evaluation of the neonate demonstrated brisk diuresis and electrolyte abnormalities. Laboratory evaluation demonstrated elevated blood urea nitrogen and creatinine levels with metabolic alkalosis, hyponatremia, and hypochloremia (Table 1).

**Table 1:** Neonatal laboratory findings supportive of suspected antenatal Bartter syndrome. Postnatal laboratory evaluation demonstrated elevated BUN and creatinine levels with metabolic alkalosis, with bicarbonate levels up to 37 mmol/L, accompanied by hyponatremia and hypochloremia. These findings were supportive of suspected antenatal Bartter syndrome. BUN: Blood urea nitrogen; mmol/L: Millimoles per liter; mg/dL: Milligrams per deciliter.

Laboratory test	Observed value range	Reference range
Sodium	124-137 mmol/L	135-145 mmol/L
Chloride	84-99 mmol/L	95-110 mmol/L
Potassium	3.9-5.5 mmol/L	3.5-6.0 mmol/L
Arterial pH	7.41-7.62	7.35-7.45
Bicarbonate	31-37 mmol/L	23-29 mmol/L
BUN	49-72 mg/dL	3-13 mg/dL
Creatinine	1.30-2.60 mg/dL	0.12-1.06 mg/dL

Additional urine studies demonstrated elevated urine sodium and a markedly increased urine calcium-to-creatinine ratio, findings suggestive of renal salt wasting and hypercalciuria (Table 2).

**Table 2:** Neonatal laboratory evaluation demonstrating electrolyte abnormalities consistent with renal salt wasting in suspected antenatal Bartter syndrome. mg/mg: Mass ratio; mg/dL: Milligrams per deciliter; Ca: Calcium; Cr: Creatinine.

Laboratory test	Observed value	Reference range
Urine sodium	89 mEq/L	<20 to 40 mEq/L
Urine calcium	11.3 mg/dL	<10 mg/dL
Urine creatinine	5.2 mg/dL	2-20 mg/dL
Urine Ca/Cr ratio	2.17 mg/mg	<0.8 to 1.0 mg/mg

**Figure 2:**
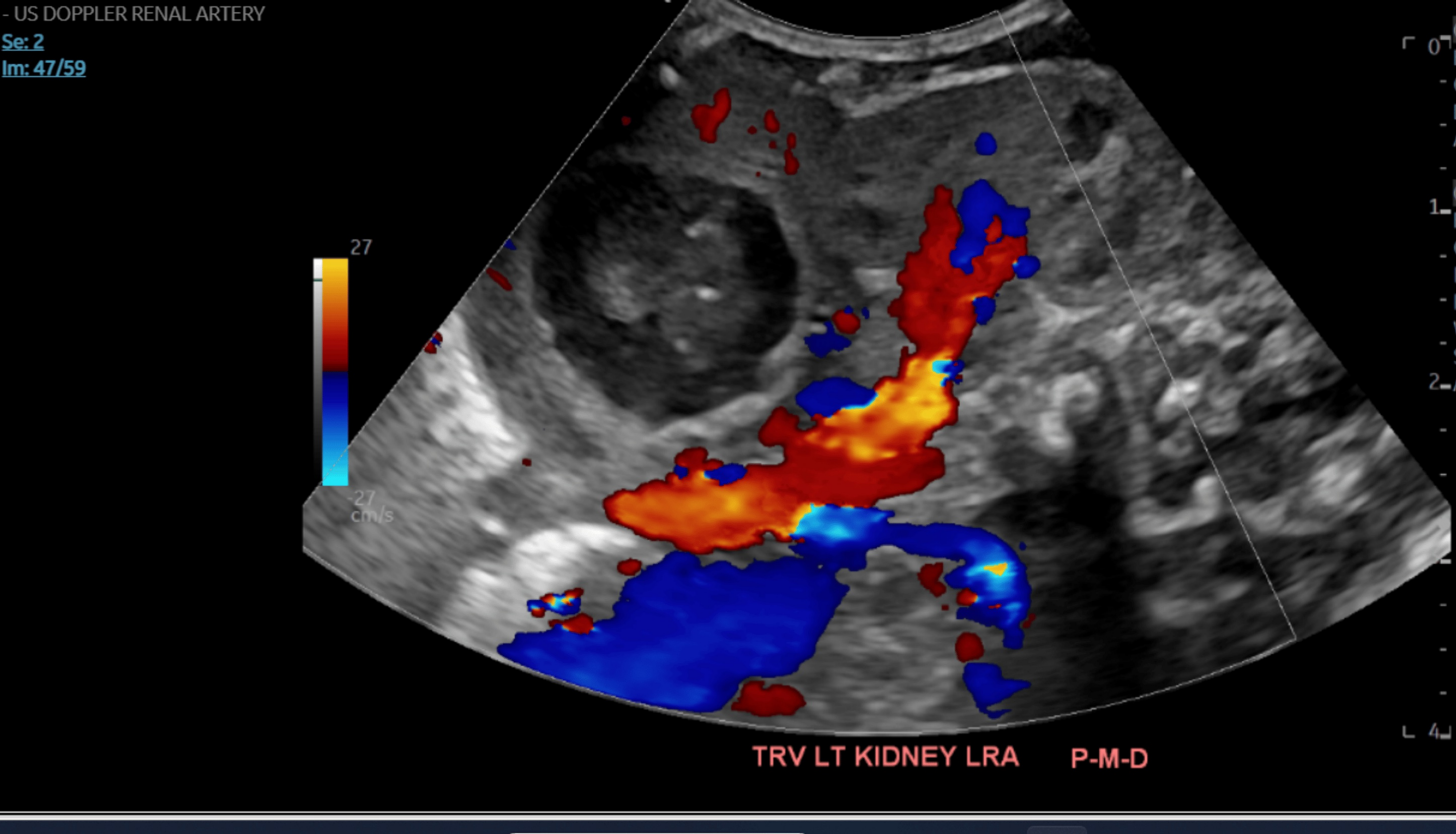
Neonatal renal Doppler ultrasound of the left kidney. Color Doppler ultrasound of the left kidney in transverse view demonstrating preserved flow within the left renal artery and intrarenal vasculature.

**Figure 3:**
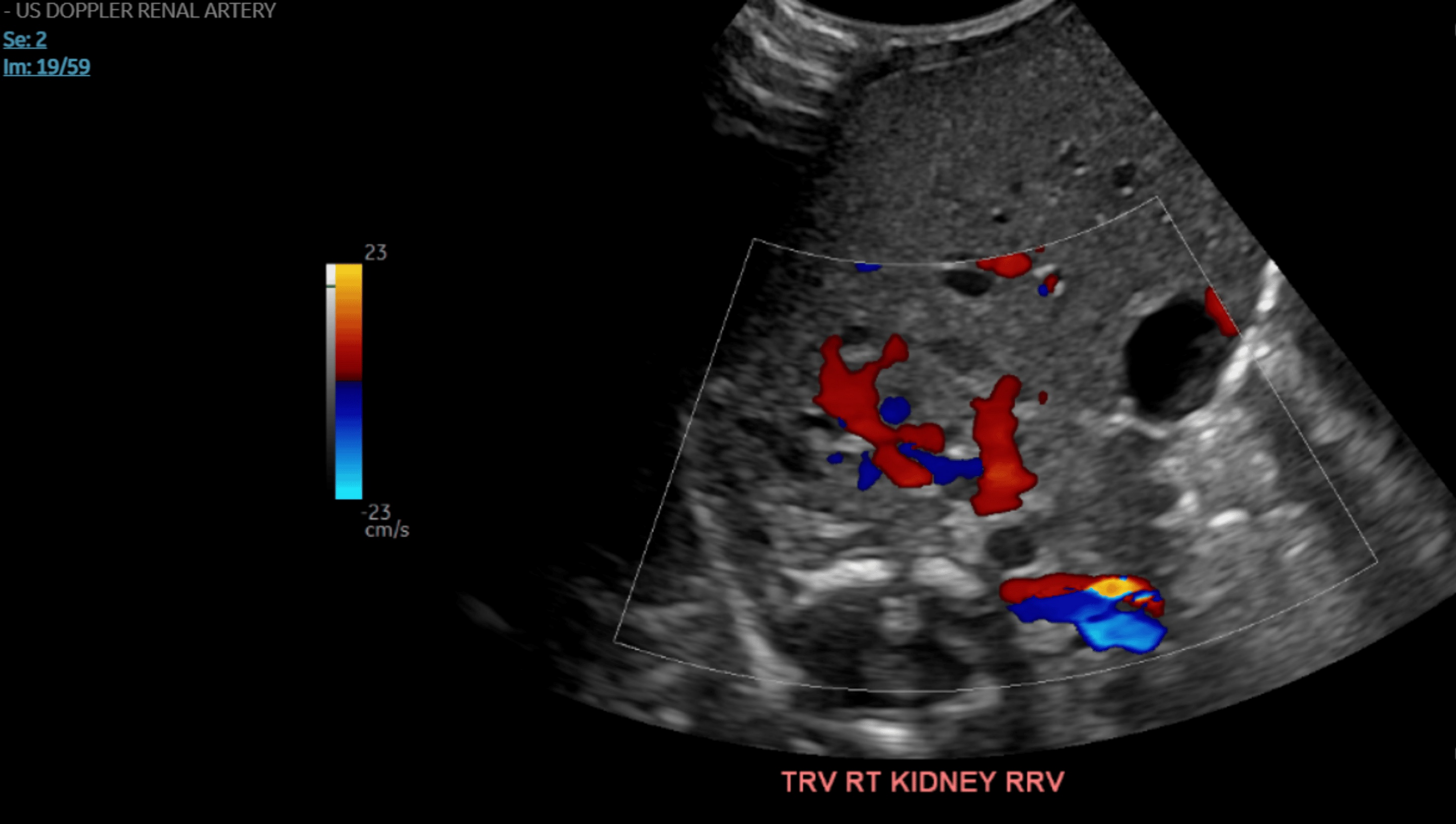
Neonatal renal Doppler ultrasound of the right kidney. Color Doppler ultrasound of the right kidney in transverse view demonstrating preserved renal hilar and intrarenal blood flow.

Renal USG with Doppler evaluation of the neonate was performed and demonstrated normal renal morphology with preserved intrarenal arterial flow and no sonographic evidence of renal artery stenosis (Figures 2-3).

Given the history of severe unexplained polyhydramnios in the setting of a structurally normal fetus, the clinical presentation and associated laboratory and ultrasonographic findings raised concern for antenatal Bartter syndrome. The neonate was managed with fluid and electrolyte replacement and subsequently transferred to a tertiary care center for confirmatory evaluation and specialized neonatal management.

## Discussion

Bartter syndrome is a rare group of inherited autosomal recessive renal tubular disorders affecting sodium chloride reabsorption in the thick ascending limb (TAL) of the loop of Henle, leading to salt wasting, fetal polyuria, severe polyhydramnios, and an increased risk of preterm delivery [1,4]. The prevalence of Bartter syndrome is estimated to be approximately 1 in 1,000,000 [4]. Major phenotypic forms include antenatal Bartter syndrome (Types I, II, and IV), classic Bartter syndrome (Type III), and Gitelman syndrome [4,5].

Antenatal Bartter syndrome represents a severe form of Bartter syndrome with onset in utero and is commonly associated with mutations in the SLC12A1 gene (Type I), which encodes the Na-K-2Cl cotransporter, and the KCNJ1 gene (Type II), which encodes the ROMK potassium channel [4]. Postnatally, affected neonates typically present with polyuria, hypokalemic metabolic alkalosis, hyperreninemia, hyperaldosteronism, nephrocalcinosis, and failure to thrive [4]. Classic Bartter syndrome generally presents during childhood and is characterized by failure to thrive, polyuria, polydipsia, muscle weakness or cramping, and growth retardation [4]. It is associated with mutations in CLCNKB, which encodes the ClC-Kb chloride channel [4]. Gitelman syndrome typically presents during late childhood, adolescence, or adulthood with hypokalemia, metabolic alkalosis, hypomagnesemia, and hypocalciuria [4,5]. Gitelman syndrome results from mutations in the calcium ion-sensing receptor (CaSR) and in the genes that encode the chloride channel subunits, ClC-Ka and ClC-Kb [5]. Hypercalciuria is characteristic of antenatal Bartter syndrome and may help distinguish it from Gitelman syndrome, which is more commonly associated with hypocalciuria [4-6].

The hallmark prenatal finding in antenatal Bartter syndrome is early-onset severe polyhydramnios in the setting of a structurally normal fetus. Polyhydramnios may result from maternal diabetes mellitus, fetal anomalies, congenital infections, alloimmunization, or may be idiopathic [2-4]. When a patient presents with unexplained polyhydramnios, a detailed medical and family history should be obtained, and USG should be performed to evaluate fetal anatomy and placental abnormalities. Severe polyhydramnios is associated with an increased risk of fetal abnormalities, which have been reported in approximately 20-40% of cases [2]. Therefore, fetal structural abnormalities, particularly gastrointestinal anomalies such as esophageal atresia and tracheoesophageal fistula, as well as renal abnormalities, should be evaluated when assessing causes of elevated amniotic fluid volume during pregnancy [2,3,7]. Following delivery, postnatal renal USG may identify nephrocalcinosis and structural renal abnormalities that can support the diagnosis, guide genotype prediction, and inform long-term management. Placental abnormalities may contribute through mechanisms such as fetal anemia and stress, which can lead to increased cardiac output and enhanced fetal renal perfusion, ultimately resulting in excessive fetal urine production [2,7].

When antenatal Bartter syndrome is suspected prenatally, amniocentesis may aid diagnosis through biochemical analysis of amniotic fluid and genetic evaluation using targeted sequencing. Abnormal amniotic fluid electrolyte profiles may raise suspicion for an underlying ion-channel or renal tubular transport disorder [1]. Molecular genetic testing remains the definitive method for confirming the diagnosis of antenatal Bartter syndrome through identification of pathogenic variants in associated ion transport genes [4-6]. In the present case, severe polyhydramnios identified on prenatal USG raised concern for antenatal Bartter syndrome and prompted further diagnostic evaluation. Although amniocentesis was offered and ultimately declined, amniotic fluid analysis could have facilitated assessment of chloride, renin, and aldosterone levels, as well as targeted genetic testing for associated mutations [2,4,6].

If antenatal Bartter syndrome is clinically suspected or confirmed, maternal indomethacin at a dose of 1.0-3.0 mg/kg/day or approximately 25 mg orally every 6 hours (100 mg/day) has been described as a therapeutic strategy to reduce amniotic fluid volume [8,9]. Indomethacin may reduce amniotic fluid volume through two proposed mechanisms: suppression of excessive fetal renal prostaglandin E2 (PGE2) production and reduction of fetal renal blood flow and urine output [10]. In this case, indomethacin was administered for tocolysis in the setting of preterm labor and was not used specifically to reduce amniotic fluid volume, as delivery occurred after completion of antenatal corticosteroid therapy. However, if amniocentesis had been pursued for confirmatory evaluation of antenatal Bartter syndrome following the diagnosis of severe polyhydramnios, or if the patient had not been in preterm labor with ruptured membranes and had continued under expectant management and surveillance, maternal indomethacin therapy could have been considered. Because indomethacin may lead to premature constriction or closure of the fetal ductus arteriosus, close fetal surveillance is recommended [8]. Although the optimal monitoring interval remains unclear, serial USG may be beneficial for ongoing assessment of amniotic fluid volume, fetal growth, and fetal well-being [2]. Therapeutic amnioreduction may also be considered for symptomatic maternal relief, particularly in patients experiencing dyspnea or other complications related to severe polyhydramnios.

Postnatal management of antenatal Bartter syndrome is primarily supportive and includes fluid replacement, electrolyte correction, and multidisciplinary neonatal care. In the present case, management was guided by these principles, with initiation of fluid and electrolyte replacement and transfer to a higher-level care facility for confirmatory evaluation and specialized neonatal management. Prognosis varies according to disease subtype and severity; however, early recognition and intervention may improve electrolyte control and neonatal outcomes. This case highlights the diagnostic complexity of severe unexplained polyhydramnios and emphasizes the importance of maintaining antenatal Bartter syndrome in the differential diagnosis when polyhydramnios occurs in the setting of a structurally normal fetus. Early clinical suspicion may facilitate anticipatory neonatal planning, timely electrolyte management, multidisciplinary coordination, and improved outcomes.

## Conclusions

Severe polyhydramnios presenting early in the second or third trimester of pregnancy should raise suspicion for an underlying etiology. Antenatal Bartter syndrome should be considered in cases of severe, early-onset, unexplained polyhydramnios in the setting of a structurally normal fetus. Through shared decision-making, amniocentesis may be considered to evaluate amniotic fluid for biochemical abnormalities and facilitate genetic testing for associated mutations. Early recognition may support targeted antenatal management, informed counseling, and appropriate neonatal preparation. Although rare, prompt recognition of this condition may improve maternal and neonatal outcomes through coordinated obstetric and neonatal care.
